# Supplementary material for: Effects of Long-Term Fasting on Gut Microbiota, Serum Metabolome, and Their Association in Male Adults
Source: Nutrients. 2024 Dec 26;17(1):35. doi: 10.3390/nu17010035 (PMC11722564; doi:10.3390/nu17010035)
Supplement: Supplementary file 1 [file nutrients-17-00035-s001.zip › nutrients-3353358-supplementary.pdf]

Supplementary Materials

Supplementary Table S1: The diet during the calorie restriction period

	CR1	CR2	CR3	CR4
Breakfast	Rice soup	Porphyra egg soup	Millet porridge	Porphyra egg soup,<br>A piece of bread
Lunch	Egg soup	Noodle	Rotten rice, Tomato egg, Minced beef thick soup	Rice, 2 dishes of vegetables, Chicken soup
Dinner	Cabbage egg soup	Corn egg porridge	Corn egg porridge,<br>Steamed bread	Millet porridge or rice, 1 dishes of vegetable, Beef soup

Vegetable are celery, broccoliwomen,Cabbage, carrots etc.

Supplementary Table S2: The correction between differential gut microbiota and fat acylcarnitines and tryptophan derivative metabolites during prolonged complete fasting (|r|>0.6, P<0.05)

Carnitine related metabolites	Gut microbiota	Spearman correlation	P value
Hexadec-2-enoyl-carnitine	Ruthenibacterium lactatiformans	0.7687	4.5028E-11
Vaccenyl-carnitine	Ruthenibacterium lactatiformans	0.7605	9.5595E-11
L-Palmitoylcarnitine	Ruthenibacterium lactatiformans	0.7436	4.0726E-10
L-Acetylcarnitine	Ruthenibacterium lactatiformans	0.7364	7.3428E-10
5-Tetradecenoylcarnitine	Ruthenibacterium lactatiformans	0.7312	1.1047E-09
Hexadec-2-enoyl-carnitine	Escherichia coli	0.7221	2.2276E-09
Hexadec-2-enoyl-carnitine	Parvimonas micra	0.7115	4.8553E-09
Hexadec-2-enoyl-carnitine	Flavonifractor plautii	0.7099	5.4540E-09
5-Tetradecenoylcarnitine	Flavonifractor plautii	0.7088	5.8928E-09
Vaccenyl-carnitine	Alistipes indistinctus	0.7035	8.5501E-09
Linoelaidyl-carnitine	Ruthenibacterium lactatiformans	0.6993	1.1430E-08
Tetradecanoylcarnitine	Ruthenibacterium lactatiformans	0.6950	1.5251E-08
5-Tetradecenoylcarnitine	Parvimonas micra	0.6881	2.4114E-08
Linoleyl-carnitine	Ruthenibacterium lactatiformans	0.6871	2.5828E-08
Vaccenyl-carnitine	Escherichia coli	0.6863	2.7073E-08
Hexadec-2-enoyl-carnitine	Hungatella hathewayi	0.6806	3.9040E-08
3-Methylglutarylcarnitine	Ruthenibacterium lactatiformans	0.6802	4.0177E-08
Vaccenyl-carnitine	Flavonifractor plautii	0.6798	4.1109E-08
Linoleyl-carnitine	Escherichia coli	0.6796	4.1582E-08
L-Acetylcarnitine	Alistipes indistinctus	0.6731	6.2689E-08
2-Methylbutyroylcarnitine	Anaerotignum neopropionicum	0.6717	6.8142E-08
5-Tetradecenoylcarnitine	Escherichia coli	0.6713	6.9818E-08
Vaccenyl-carnitine	Parvimonas micra	0.6676	8.7381E-08
L-Hexanoylcarnitine	Flavonifractor plautii	0.6595	1.4202E-07
L-Hexanoylcarnitine	Ruthenibacterium lactatiformans	0.6585	1.5053E-07
L-Palmitoylcarnitine	Alistipes indistinctus	0.6539	1.9634E-07
5-Tetradecenoylcarnitine	Hungatella hathewayi	0.6535	2.0074E-07
L-Acetylcarnitine	Escherichia coli	0.6529	2.0707E-07
Hexadec-2-enoyl-carnitine	Alistipes indistinctus	0.6492	2.5663E-07
L-Acetylcarnitine	Anaerotruncus colihominis	0.6479	2.7593E-07
L-Acetylcarnitine	Parvimonas micra	0.6459	3.0817E-07
Hexadec-2-enoyl-carnitine	Anaerostipes caccae	0.6453	3.1833E-07
L-Palmitoylcarnitine	Parvimonas micra	0.6425	3.7278E-07

Tetradecanoylcarnitine	Parvimonas micra	0.6387	4.5667E-07
Linoelaidyl-carnitine	Escherichia coli	0.6338	5.9741E-07
L-Acetylcarnitine	Anaeroglobus geminatus	0.6330	6.2093E-07
Tetradecanoylcarnitine	Flavonifractor plautii	0.6322	6.4836E-07
5-Tetradecenoylcarnitine	Anaerotruncus colihominis	0.6302	7.2032E-07
Linoelaidyl-carnitine	Eisenbergiella tayi	0.6300	7.2722E-07
Linoelaidyl-carnitine	Parvimonas micra	0.6297	7.4163E-07
L-Acetylcarnitine	Flavonifractor plautii	0.6280	8.1111E-07
Hexadec-2-enoyl-carnitine	Anaerotruncus colihominis	0.6273	8.3838E-07
2-Methylbutyroylcarnitine	Subdoligranulum variabile	0.6262	8.9130E-07
Linoelaidyl-carnitine	Subdoligranulum variabile	0.6253	9.2975E-07
Vaccenyl-carnitine	Anaerotruncus colihominis	0.6224	1.0793E-06
Dodecanoylcarnitine	Ruthenibacterium lactatiformans	0.6201	1.2171E-06
Linoleyl-carnitine	Parvimonas micra	0.6196	1.2456E-06
Linoleyl-carnitine	Anaerotruncus colihominis	0.6193	1.2684E-06
L-Acetylcarnitine	Anaerostipes caccae	0.6168	1.4374E-06
L-Palmitoylcarnitine	Escherichia coli	0.6166	1.4547E-06
Vaccenyl-carnitine	Anaeroglobus geminatus	0.6136	1.6887E-06
Tetradecanoylcarnitine	Hungatella hathewayi	0.6118	1.8471E-06
3-Methylglutarylcarnitine	Alistipes indistinctus	0.6070	2.3360E-06
Linoelaidyl-carnitine	Alistipes indistinctus	0.6057	2.4840E-06
Tetradecanoylcarnitine	Escherichia coli	0.6049	2.5841E-06
L-Hexanoylcarnitine	Parvimonas micra	0.6028	2.8612E-06
L-Hexanoylcarnitine	Escherichia coli	0.6024	2.9059E-06
L-Hexanoylcarnitine	Subdoligranulum variabile	0.6002	3.2368E-06
Linoleyl-carnitine	Veillonella parvula	-0.6062	2.4241E-06
Tetradecanoylcarnitine	Veillonella rodentium	-0.6095	2.0613E-06
L-Palmitoylcarnitine	Veillonella parvula	-0.6112	1.9006E-06
L-Acetylcarnitine	Coprococcus comes	-0.6323	6.4526E-07
Linoelaidyl-carnitine	Veillonella parvula	-0.6429	3.6459E-07
5-Tetradecenoylcarnitine	Veillonella parvula	-0.6459	3.0898E-07
Hexadec-2-enoyl-carnitine	Veillonella parvula	-0.6568	1.6580E-07
Tetradecanoylcarnitine	Veillonella parvula	-0.6715	6.9174E-08
Indoline	Ruthenibacterium lactatiformans	0.6454	3.16298E-07
Indoline	Anaeroglobus geminatus	0.6011	3.09877E-06
Indoline	Clostridiumin nocuum	0.6351	5.55587E-07
Indoline	Clostridium methylpentosum	0.6302	7.20321E-07
Indoline	Mogibacterium diversum	0.6189	1.29528E-06
Indoline	Parvimonas micra	0.6399	4.29062E-07
Indolelactic acid	Ruthenibacterium lactatiformans	0.7332	9.44636E-10
Indolelactic acid	Flavonifractor plautii	0.6732	6.21193E-08
Indolelactic acid	Hungatella hathewayi	0.6028	2.85599E-06
Indoxyl_sulfate	Anaeroglobus geminatus	-0.6129	1.74993E-06
Indoxyl_sulfate	Escherichia coli	-0.6067	2.36736E-06
Indole_3_propionic_acid	Anaeroglobus geminatus	-0.6507	2.35011E-07
Indole_3_propionic_acid	Anaerostipes caccae	-0.6688	8.13786E-08
Indole_3_propionic_acid	Clostridium bolteae	-0.6483	2.69008E-07

Indole_3_propionic_acid	Clostridium citroniae	-0.7061	7.1398E-09
Indole_3_propionic_acid	Hungatella hathewayi	-0.6182	1.34009E-06

Supplementary Table S3: The correction between the top 5 counts of biochemical indexes and differential gut microbiota during prolonged complete fasting

Biochemical index	Gut microbiota	Spearman correlation	P value
Uric acid	Ruthenibacterium lactatiformans	0.7330	9.60E-10
DBiL	Anaerostipes caccae	0.7087	5.92E-09
CHOL	Intestinimonas butyriciproducens	0.6919	1.88E-08
LDL-C	Intestinimonas butyriciproducens	0.6893	2.23E-08
IBiL	Flavonifractor plautii	0.6864	2.69E-08
TBiL	Flavonifractor plautii	0.6809	3.83E-08
CHOL	Parabacteroides goldsteinii	0.6761	5.19E-08
IBiL	Anaerotruncus colihominis	0.6730	6.29E-08
T3	Coprococcus comes	0.6722	6.59E-08
DBiL	Anaeroglobus geminatus	0.6599	1.38E-07
IBiL	Ruthenibacterium lactatiformans	0.6589	1.47E-07
Vit A	Ruthenibacterium lactatiformans	0.6589	1.47E-07
Cre	Catabacter hongkongensis	0.6582	1.53E-07
FT3	Anaerostipes caccae	-0.6557	1.77E-07
TBiL	Ruthenibacterium lactatiformans	0.6527	2.10E-07
Uric acid	Eisenbergiella tayi	0.6516	2.23E-07
T3	Anaerostipes caccae	-0.6504	2.40E-07
TBiL	Anaerotruncus colihominis	0.6472	2.86E-07
LDL-C	Parabacteroides goldsteinii	0.6358	5.34E-07
Uric acid	Clostridium citroniae	0.6352	5.54E-07
FT3	Coprococcus comes	0.6327	6.33E-07
TBiL	Anaerostipes caccae	0.6308	6.98E-07
Uric acid	Anaerotruncus colihominis	0.6304	7.15E-07
CHOL	Eisenbergiella tayi	0.6301	7.25E-07
FT3	Ruthenibacterium lactatiformans	-0.6291	7.65E-07
Uric acid	Hungatella hathewayi	0.6220	1.10E-06
LDL-C	Eisenbergiella tayi	0.6206	1.19E-06
DBiL	Flavonifractor plautii	0.6200	1.22E-06
Cre	Eisenbergiella tayi	0.6187	1.30E-06
Uric acid	Flavonifractor plautii	0.6179	1.36E-06
Uric acid	Parvimonas micra	0.6169	1.43E-06
CO2-cp	Veillonella parvula	0.6150	1.57E-06
CHOL	Collinsella aerofaciens	0.6126	1.77E-06
TBiL	Parvimonas micra	0.6118	1.85E-06
DBiL	Lachnoclostridium sp.YL32	0.6107	1.94E-06
uCRE	Ruthenibacterium lactatiformans	0.6070	2.33E-06
DBiL	Parvimonas micra	0.6063	2.41E-06
Vit A	Flavonifractor plautii	0.6059	2.46E-06
uCRE	Hungatella hathewayi	0.6053	2.54E-06
LDL-C	Pseudoflavonifractorca pillosus	0.6038	2.73E-06
LDL-C	Parvimonas micra	0.6026	2.88E-06

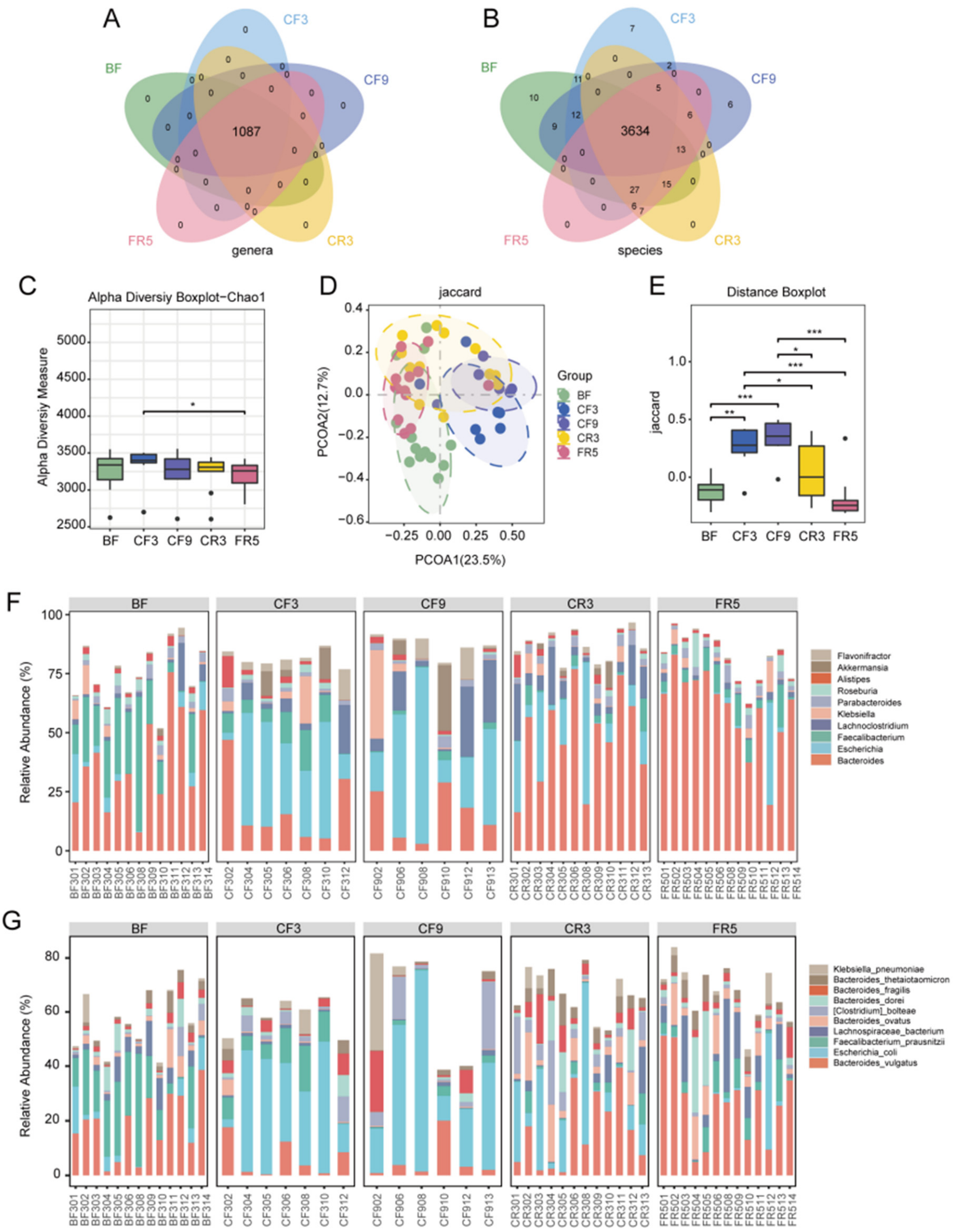

Supplementary Figure S1: 10-day complete fasting affected the diversity and composition of the human gut microbiota. Venn diagram showed the common and unique genera (A) and species (B) during the different fasting periods. (C) Compare alpha diversity based on the Chao1 index in the gut microbiota across different fasting time points. (D) Principal coordinate analysis (PCoA) plot of the gut microbiota during the fasting experiments based on Jaccard distances. (E) Boxplot showed the distribution of Jaccard's distance from samples among the different courses in the fasting experiment based on the OTU abundance. Bar plot of the relative abundance of top ten genera (F) and species (G) in the gut microbiota. Each bar represented a single participant and was labeled with a subject identifier. Boxes and whiskers showed quartiles with outliers as individual points. * P<0.05,

** P<0.01, *** P< 0.001; Significant difference (P<0.05) determined by Wilcox test (C, E). BF: before fasting; CF3: 3rd day of complete fasting; CF9: 9th day of complete fasting; CR3: 3rd day of calorie restriction; FR5: 5th day of full recovery.

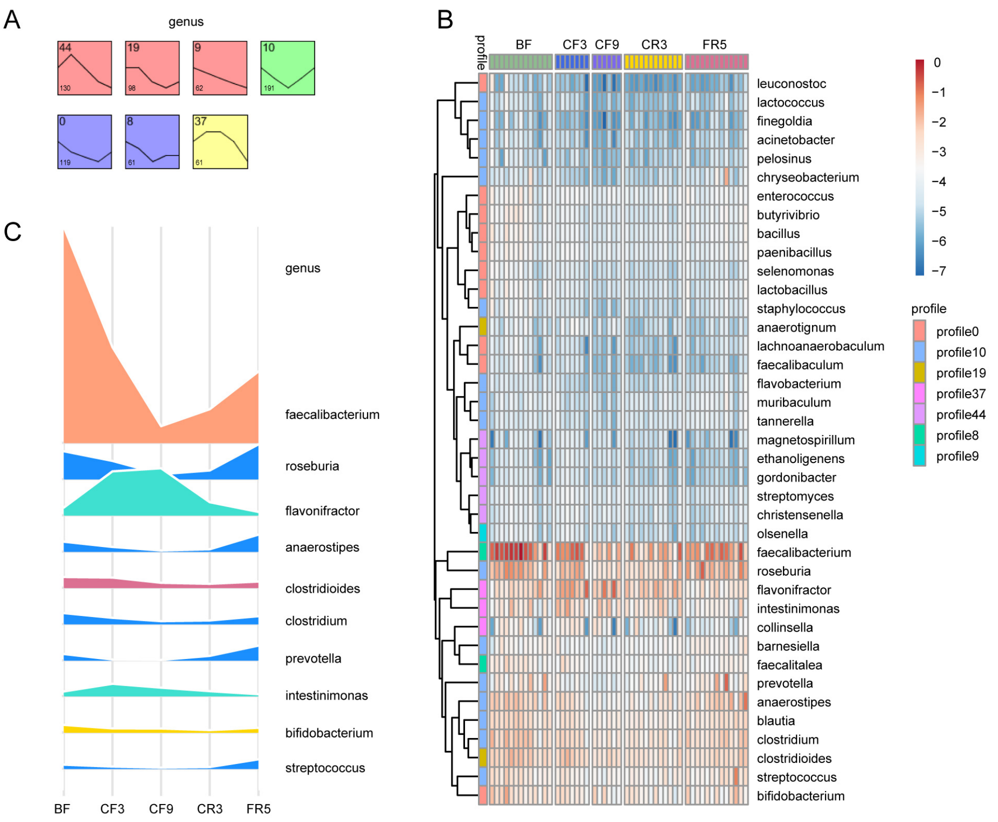

Supplementary Figure S2: 10-day complete fasting induced the different change patterns of human gut genera. (A) Patterns of genera change across the different fasting time points inferred by short time-series expression miner (STEM) analysis. Statistically significant profiles (P<0.05) were represented in color. Similar colors represented the same type of change profile. The upper left number was the profile ID and the lower left number presented the species count in each box. (B) Heatmap of the relative abundance of the genera with significant difference (based on the Permutation test) using log10(X+min(X [X!=0]) (X: the relative abundance of the genera)) by R with colors gradually changing from blue to red, corresponding to low and high relative abundance, respectively and trend (based on the STEM analysis) (P<0.05) over the time course of the fasting experiment. (C) The ridgeline plot showing the top 10 most abundant genera in the heatmap. A significant difference (P< 0.05) was determined by the Permutation test (B). BF: before fasting; CF3: 3rd day of complete fasting; CF9: 9th day of complete fasting; CR3: 3rd day of calorie restriction; FR5: 5th day of full recovery.

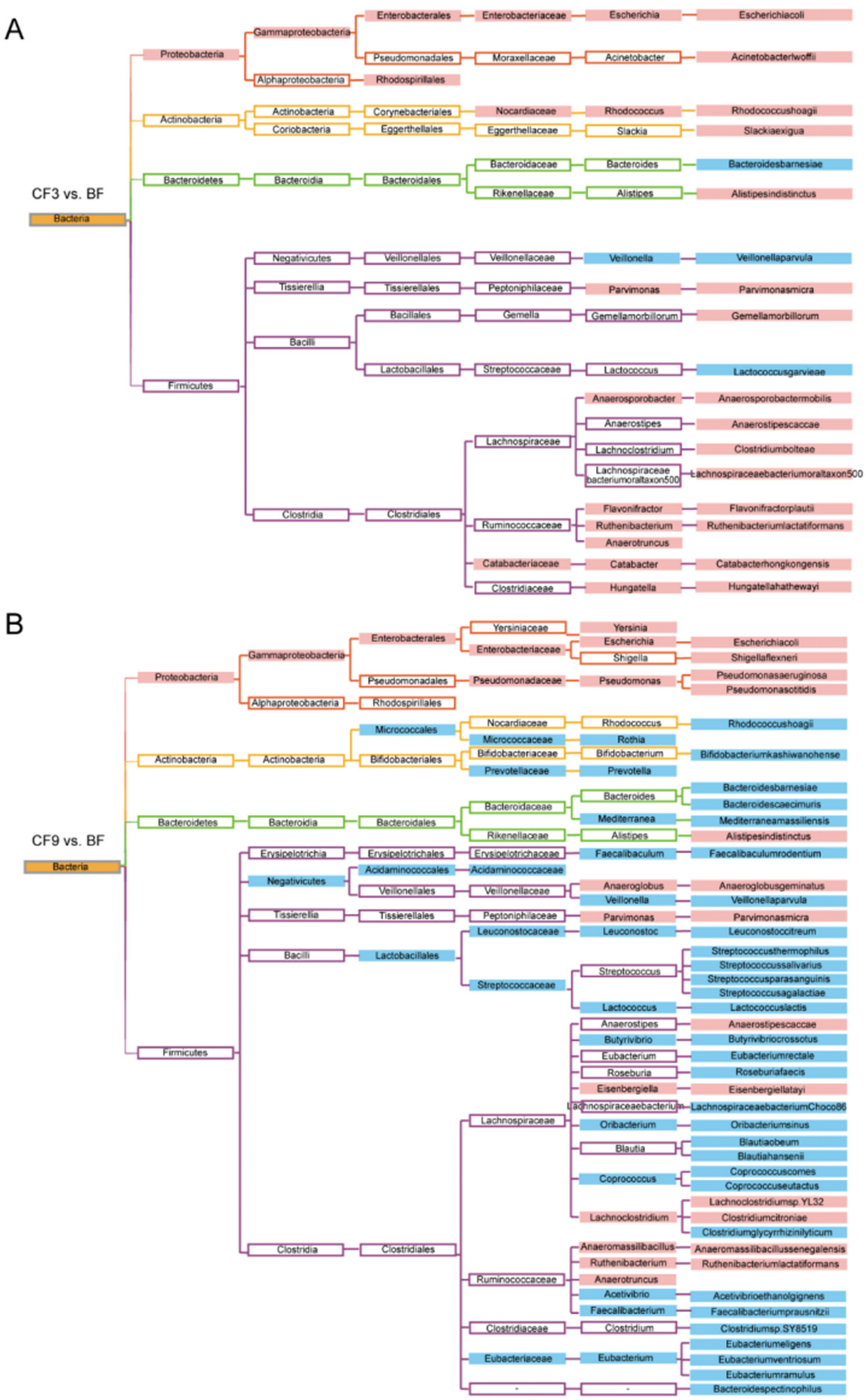

Supplementary Figure S3: The relative abundance of human gut microbiota was significantly affected at CF3 and CF9. The significantly changed gut bacteria at CF3 (A) and CF9 (B) compared with BF by Dunn test. The pink fill represented an increased trend, and the blue fill represented a decreased trend. BF: before fasting; CF3: 3rd day of complete fasting; CF9: 9th day of complete fasting.

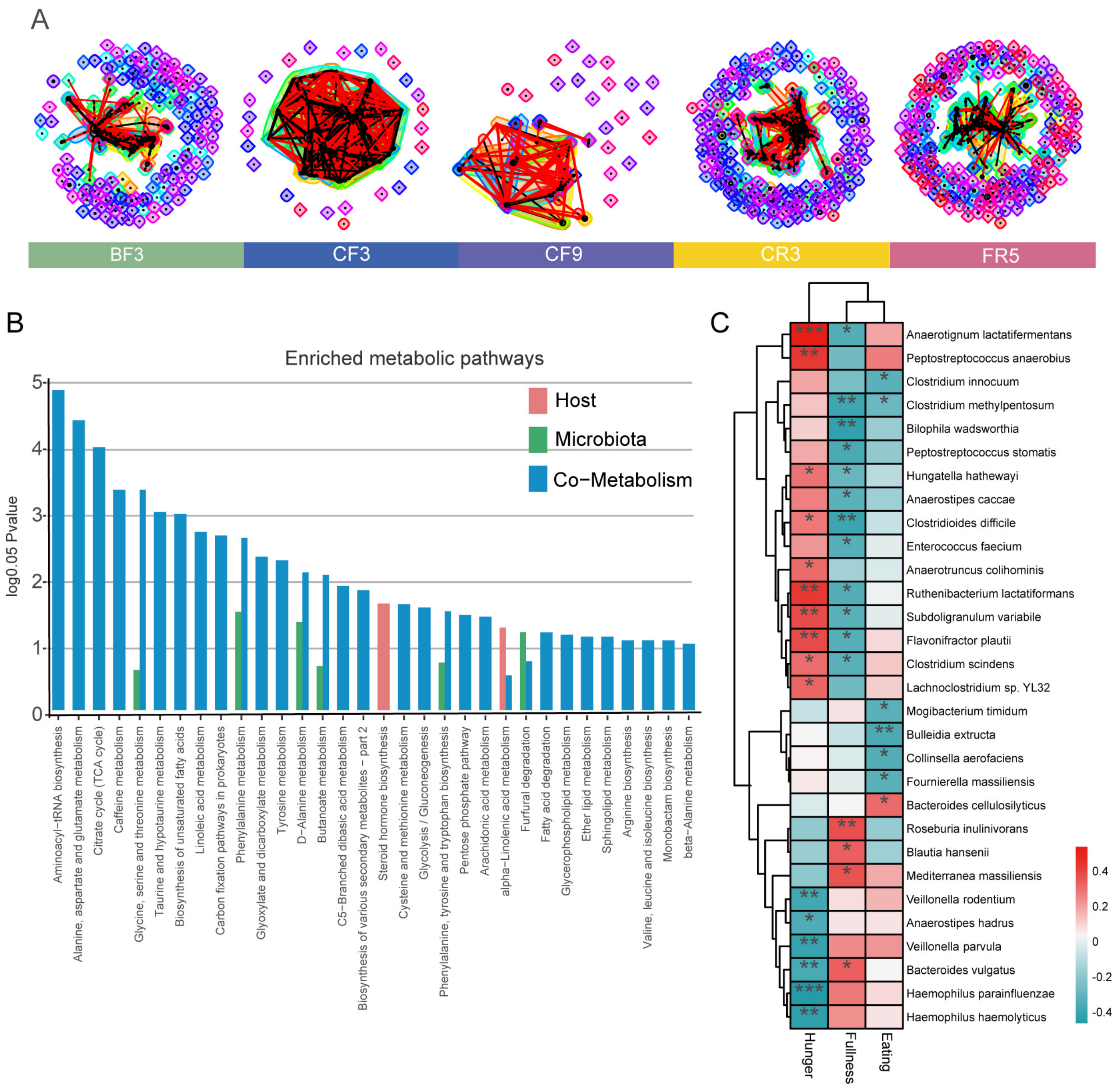

Supplementary Figure S4: The effects of 10-day fasting on the topological structure of the microbial interaction networks (|r|≧0.8 and P<0.05) at different time points (A); on the metabolic pathways of metabolites from the host, microbiota and Co-Metabolism (B) and the the correlation between microbiota and diet-related feeling (C). * P<0.05,** P<0.01, *** P<0.001.

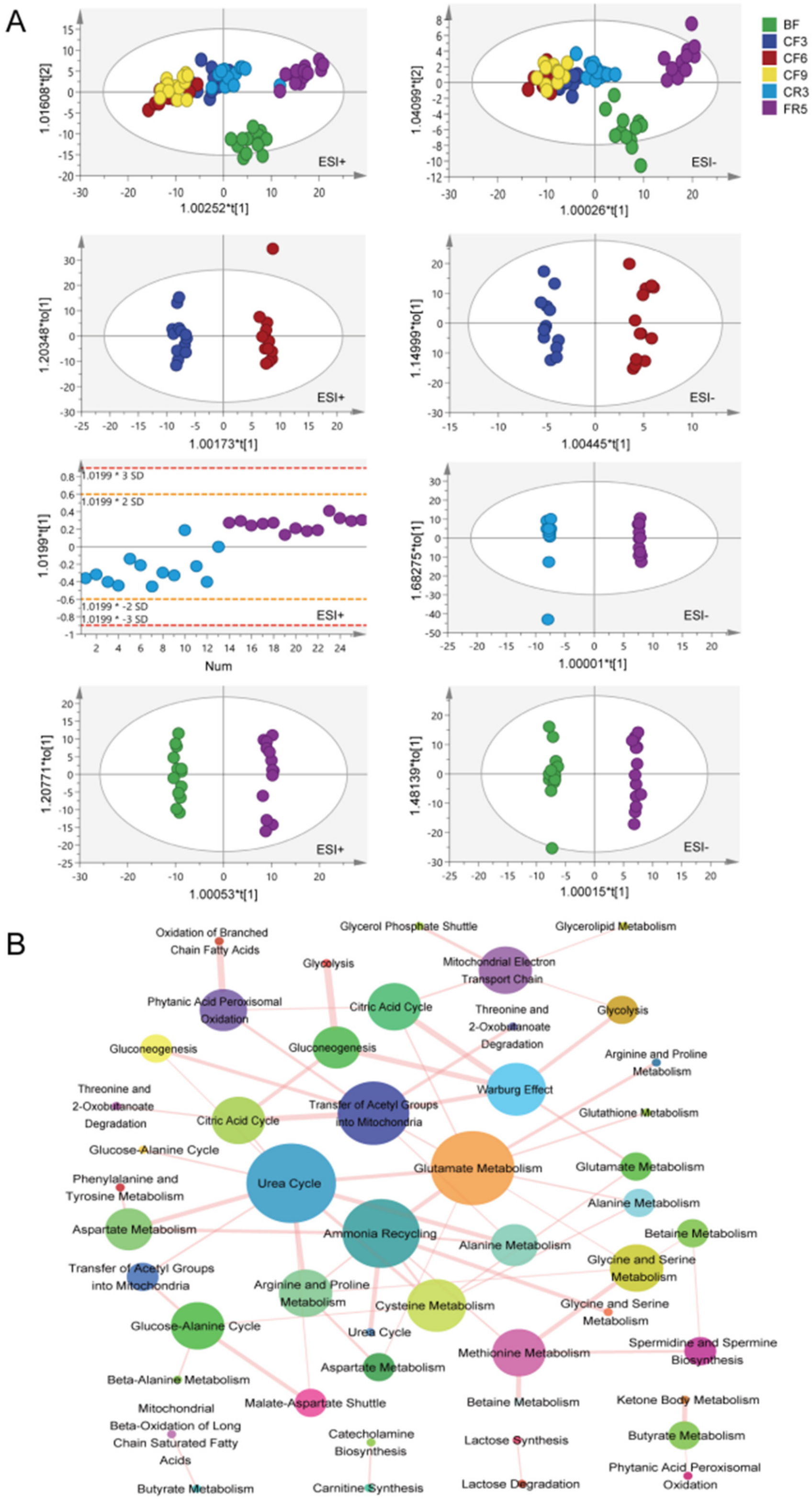

Supplementary Figure S5: Complete fasting restructured the serum metabolites and metabolic pathways. A: OPLS-DA distribution of positive (ESI+) and negative (ESI-) ions during the different fasting periods. (B) Enrichment map of metabolic pathways involved in differential metabolites. The thickness of the line represented the absolute value of the correlation coefficient. BF: before fasting; CF3: 3rd day of complete fasting; CF9: 9th day of complete fasting; CR3: 3rd day of calorie restriction; FR5: 5th day of full recovery.

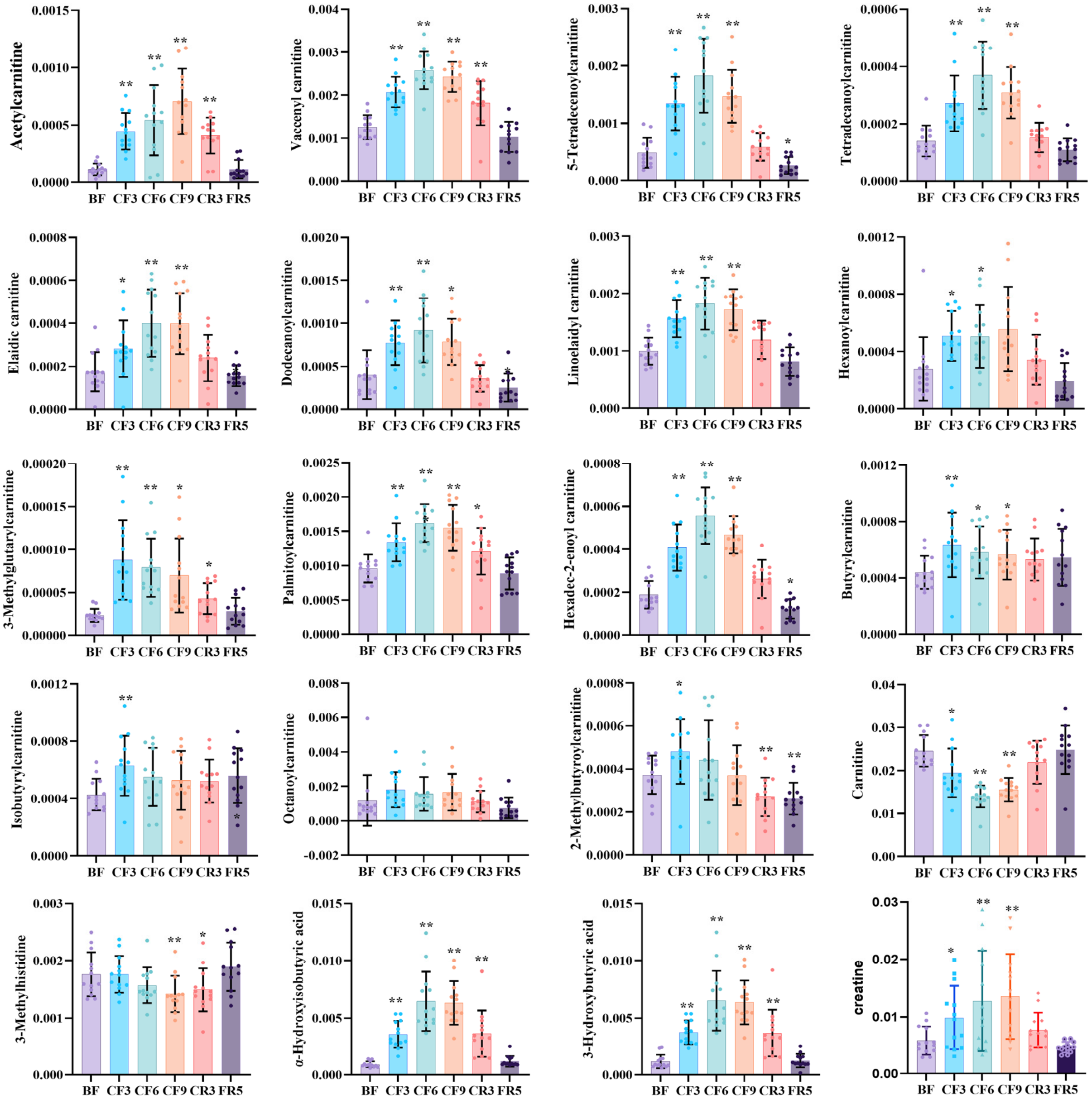

Supplementary Figure S6: The relative abundance changes of fatty acylated carnitine molecules and SCFA-associated metabolites during 10-day complete fasting. * P< 0.05, ** P<0.01, VS. BF, n=13. BF: before fasting; CF3: 3rd day of complete fasting; CF9: 9th day of complete fasting; CR3: 3rd day of calorie restriction; FR5: 5th day of Full recovery.

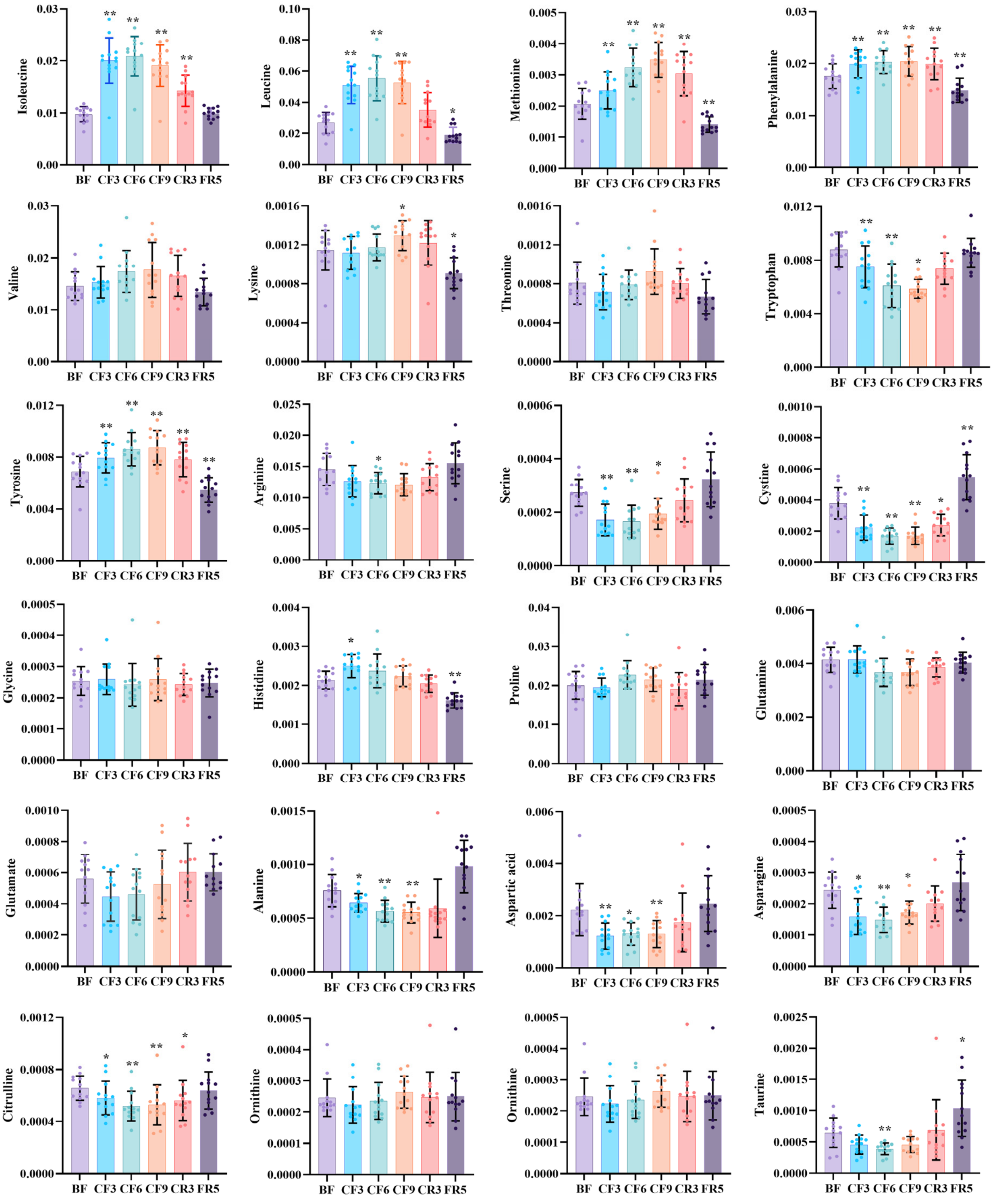

Supplementary Figure S7: The relative abundance changes of amino acids during 10-day complete fasting. * P<0.05, ** P<0.01, VS. BF, n=13. BF: before fasting; CF3: 3rd day of complete fasting; CF9: 9th day of complete fasting; CR3: 3rd day of calorie restriction; FR5: 5th day of full recovery.

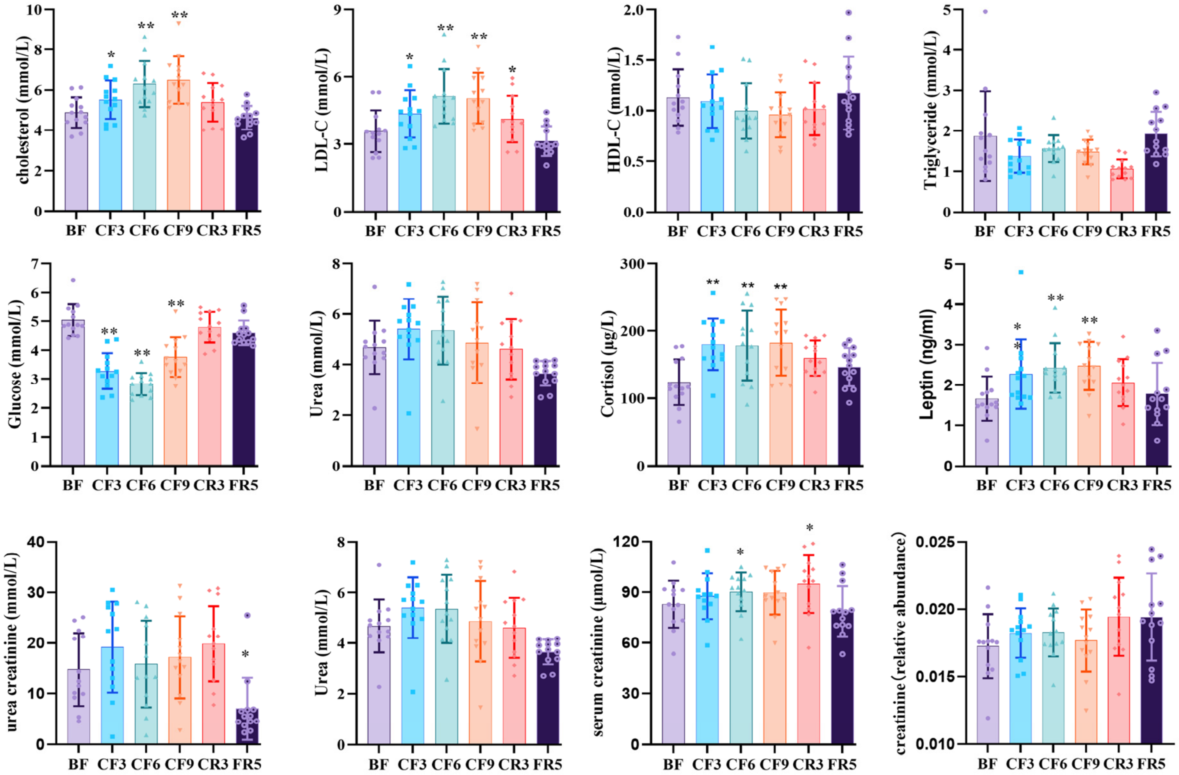

Supplementary Figure S8: The changes in fat metabolism relative to biochemical indexes during 10-day complete fasting (5). * P<0.05, ** P<0.01, VS. BF, n=13. BF: before fasting; CF3: 3rd day of complete fasting; CF9: 9th day of complete fasting; CR3: 3rd day of calorie restriction; FR5: 5th day of full recovery.

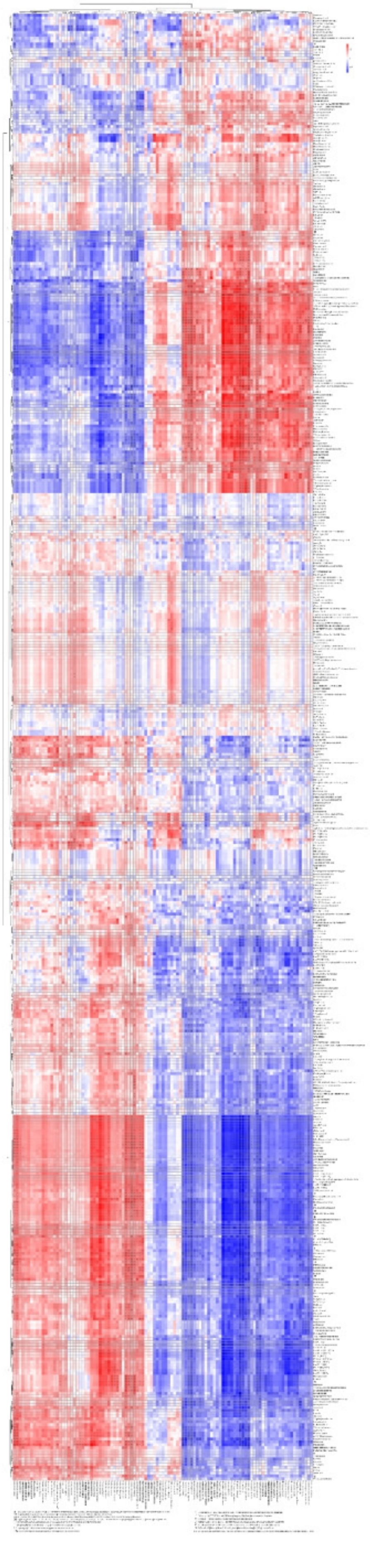

Supplementary Figure S9: The correction between differential metabolites and gut microbiota during 10-day complete fasting.

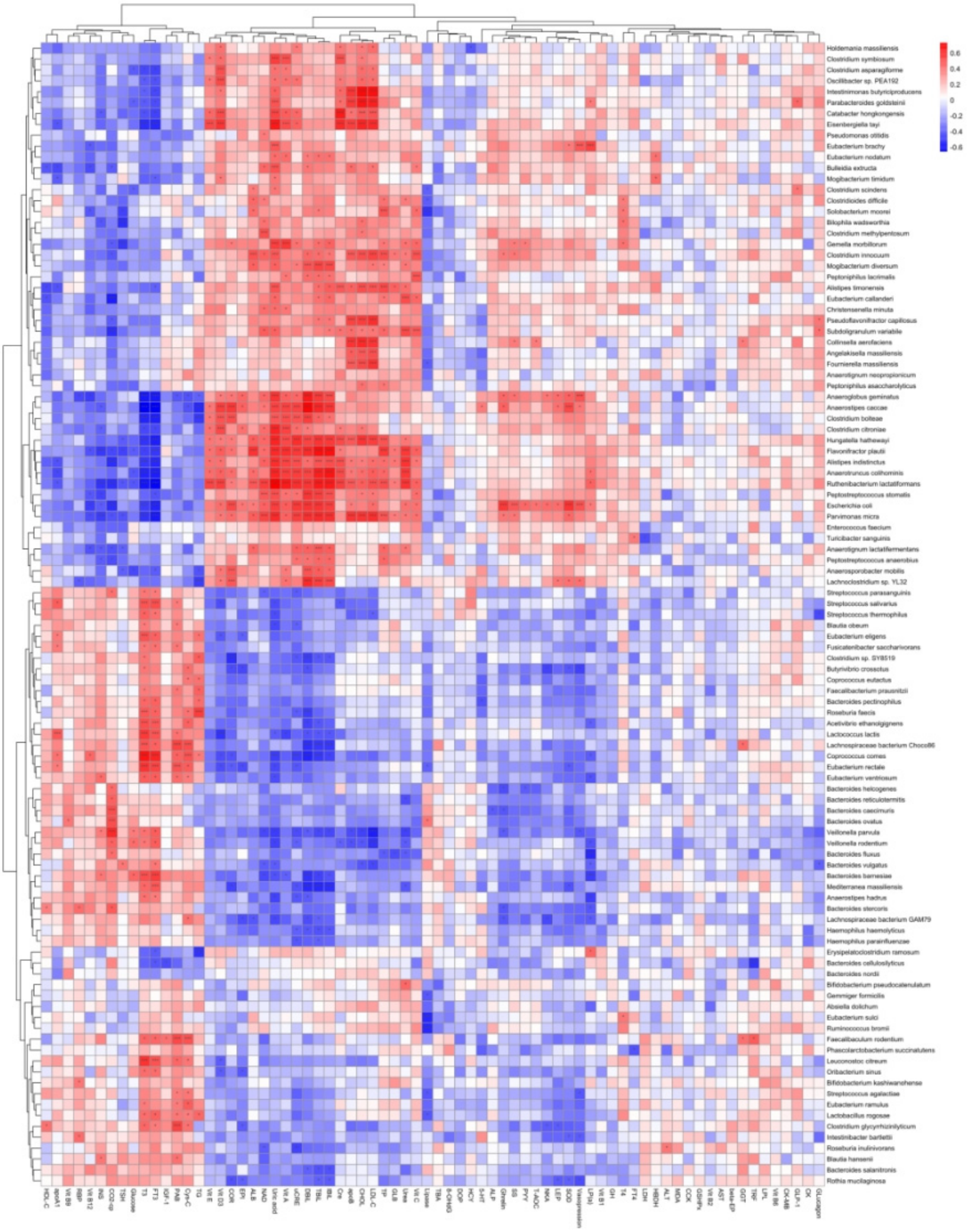

Supplementary Figure S10: The correction between differential gut microbiota and fat metabolism relative biochemical indexes during 10-day complete fasting.
